# Extracts from *Vatica diospyroides* Type SS Fruit Show Low Dose Activity against MDA-MB-468 Breast Cancer Cell-Line via Apoptotic Action

**DOI:** 10.1155/2014/479602

**Published:** 2014-08-28

**Authors:** Theera Srisawat, Yaowapa Sukpondma, Siriphon Chimplee, Kanyanatt Kanokwiroon, Aman Tedasen, Potchanapond Graidist

**Affiliations:** ^1^Faculty of Science and Industrial Technology, Prince of Songkla University, Surat Thani Campus, Surat Thani 84000, Thailand; ^2^Department of Chemistry, Faculty of Science, Prince of Songkla University, Hat Yai, Songkhla 90110, Thailand; ^3^Department of Biomedical Sciences, Faculty of Medicine, Hat Yai, Songkhla 90110, Thailand; ^4^The Excellent Research Laboratory of Cancer Molecular Biology, Prince of Songkla University, Hat Yai, Songkhla 90110, Thailand

## Abstract

Very strong antiproliferative action of *V. diospyroides* type SS fruit extracts (IC_50_ range of 1.60-17.45 *µ*g/mL) in MDA-MB-468 cell-line was observed in an MTT assay. After dosing of an extract concentration at half IC_50_ to cell line for 24 to 72 hours, treated cells were subjected to Annexin V-FITC/PI binding assay, followed by FACS and western blot analyses. Significant apoptotic death was observed with all extract treatments and both exposure times. Dosing with acetone extract of pericarp and cotyledon induced the highest apoptotic populations (33 and 32%, resp.), with the lowest populations of viable cells (65 and 67%, resp.). During 24 to 72 hours of dosing with methanolic extract of pericarp, the populations of viable and early apoptotic cells decreased significantly from 72.40 to 71.32% and from 12.00 to 6.36%, respectively, while the late apoptotic and nonviable cell populations continuously increased from 15.30 to 19.18% and from 0.30 to 3.14%, respectively. The expression of Bax increased within 12–48 hours of dosing, confirming apoptosis induced by time-dependent responses. The mutant p53 of MDA-MB-468 cells was expressed. Our results indicate that apoptosis and time-dependent therapeutic actions contribute to the cytotoxic effects of *V. diospyroides* type SS fruit on MDA-MB-468 cell.

## 1. Introduction

At present, breast cancer is commonly considered the globally leading cancer type that contributes to mortality of women. The types of breast cancer are categorized based on estrogen level and on responsiveness of the cancer cells. The estrogen-dependent types (ER-rich), such as the MCF-7 cell-line, are responsive to chemotherapy, while the estrogen-independent types (ER-poor), such as the MDA-MB-231 cell-line, are aggressive unresponsive drug-resistant cancers [1, 2]. The therapeutic intervention against a breast cancer usually comprises both chemoradiation therapy and surgical operations, but the survival rates and times remain very low [3] and depend on the type and the stage of cancer. Moreover, patients with the ER-poor type of breast cancer may suffer from negative toxic effects caused by high drug doses. The dose level of a drug can induce various modes of breast cancer cell death, with apoptosis being dominant at a low dose level in both ER-rich and ER-poor types and necrosis being dominant in the ER-rich type at a high dose level [4, 5]. Only apoptosis is effective and it is critical for the success of a cancer treatment. Therefore, much of current research seeks to discover natural drugs that would be effective and nontoxic at a low dose level [2], for inducing apoptosis of breast cancer both* in vitro* and* in vivo*. Such programs mostly focus on medicinal plants as sources of the active ingredients.

*Vatica diospyroides* Symington, a plant belonging to the family Dipterocarpaceae, is an important source of breast cancer chemopreventives, such as phytochemical constituents like saponins and terpenoids components [6], and resveratrol tetramer [7, 8]. The resveratrol derivatives like dibalanocarpol and vaticaffinol purified from plants in this family have been conducted as several biological properties against HIV and funguses [9, 10]. Interestingly, the resveratrol tetramer like vaticanol and vaticaphenol series that play an important role as the major antineoplastic constituent has been purified from both stem and bark of many Dipterocarpaceae plants. Based on* in vitro* breast cancer experiments using* V. diospyroides* fruit, these are a better source of therapeutic agents with antiproliferative effects than the other plant parts such as root [6, 11]. In our previous work,* V. diospyroides* type SS fruit had antiproliferative action and induced apoptosis and/or necrosis, depending on the extract concentration used, in MCF-7 and MDA-MB-231 breast cancer cell-lines [5]. Aside from dependence on the dose level, the inhibition via apoptotic pathways of ER-poor MDA-MB-468 cell-line, an* in vitro* model of malignant and invasive breast cancer, also depends on the duration of incubation [2]. Therefore, in this study, dose levels below the IC_50_ of a* V. diospyroides* type SS fruit extract could also lead to apoptosis in MDA-MB-468 cells, given time for such therapeutic action. We demonstrate the* in vitro* efficacy of* V. diospyroides* type SS fruit extract as a breast cancer chemopreventive, with antiproliferative and apoptotic effects via p53 and Bax protein related mechanisms on an ER-poor breast cancer model.

## 2. Materials and Methods

### 2.1. Plant Materials and Extract Preparations

The collection of fruit samples from ten-year-old* V. diospyroides* type SS (Collector number T. Srisawat 002) and the preparation of acetone and methanolic extracts from the fruit followed methods described previously [6]. Extracts of fruit pericarp and cotyledon were kept separate and stored in darkness at 4°C prior to tests against breast cancer (MDA-MB-468 cell, ATCC HTB-132) and normal (Vero cell, ATCC CCL-81) cell-lines in a cytotoxicity assay.

### 2.2. Cytotoxicity Assay

Two assays were performed, namely, a 3-(4,5-dimethylthiazol-2-yl)-2,5-diphenyltetrazolium bromide (MTT) assay for breast cancer cell-lines and a green fluorescence protein (GFP) assay for the normal Vero cells. For the MTT assay, the MDA-MB-468 cells were seeded in 96-well plates at a density of 2 × 10^4^ cells, in 100 *μ*L of medium per well. Each extract was diluted to the desired concentration, added into the cell culture, and then incubated for 72 hours. Adjusting to the desired 5–80 *μ*g/mL concentrations of extracts, cell viability determination and fitting of response curves, and the harvest of treated cells followed previously described methods [5]. The half-inhibitory IC_50_ concentration and the labeling of cytotoxic activity were determined according to Alitheen et al. [12] and Wibowo et al. [13] criteria, with very strong inhibiting activity meaning IC_50_ < 5 *μ*g/mL. For the normal Vero cells, the GFP assay followed the methods of Hunt et al. [14], and less than 50% cell growth indicated cytotoxicity against normal cells.

The mode of MDA-MB-468 cell death and the time of therapeutic action were determined by an Annexin V-FITC/PI binding assay and flow cytometry. To avoid necrotic cell death from acute toxicity with high extract dose levels, half IC_50_ extract concentrations were used.

### 2.3. Annexin V-FITC/PI Binding Assay

To detect apoptotic cells, the cellular location of phosphatidylserine (PS) and the cell membrane integrity/permeability of treated cells were assessed by staining and by applying Annexin V-FITC/PI binding kit, following the manufacturer's protocol (BD Pharmingen, USA). Briefly, after suspending the cells in a 1x binding buffer (0.1 M Hepes, 0.1 M NaOH pH 7.4, 1.4 M NaCl, and 25 mM CaCl_2_) at a concentration of 1 × 10^6^ cells/mL, five *μ*L each of Annexin V-FITC and propidium iodide (PI) was subsequently added. The suspensions were vortexed gently and 400 *μ*L of 1x binding buffer was added before analysis with fluorescence activated cell sorter (FACS).

### 2.4. Flow Cytometry

For FACS analysis, a FACSCalibur flow cytometer (Becton Dickinson Biosciences {BDB}, San Jose, CA), equipped with a 488 nm argon ion laser, was used. A total of 5,000 events were acquired with CellQuest software (BDB). The populations of viable, early apoptotic, late apoptotic, and dead cells in each experiment were analyzed and dot plot diagrams were generated with WinMDI version 2.9 software. The mode of cell death was determined from the transitions of cells in such plots, following criteria explained earlier [5].

### 2.5. Western Blotting

The MDA-MB-468 cells were treated with the extract at half IC_50_ (0.5 IC_50_) concentration for up to 48 hours, then released by trypsinizing the cells, and collected by centrifugation. The cells were washed once in cold PBS, prior to lyse in RIPA buffer (Peirce Biotechnology, IL, USA). Protein concentration was measured using Bradford protein assay (Biorad, CA, USA). Fifty micrograms of each protein sample was separated in 12% SDS-PAGE and electrophoretically transferred to a nitrocellulose membrane. The membranes were blocked in 5% low-fat dry milk in TBS-T (5% low-fat dry milk, 0.1% Tween-20 in Tris-buffered saline) for 1 h at room temperature. After that, each membrane was incubated overnight with primary antibodies against p53 (1 : 1000) and Bax (1 : 1000) with *β*-actin (1 : 1000) used as an internal control. All the antibodies were purchased from Cell Signaling, MA, USA. The membranes were washed 3 times (5 min/wash) with 1% low-fat dry milk in TBS-T and incubated with ECL anti-rabbit IgG horseradish peroxidase (GE health care) diluted 1 : 5000 in 1% low-fat dry milk in TBS-T, for 1 h at room temperature. After washing with TBS-T 3 times, the signals were detected using a chemiluminescent detection system (Pierce, IL, USA) and then exposed to film [5].

### 2.6. Statistical Analysis

Three independent cytotoxicity experiments were performed, and the results are expressed as mean ± SD. Statistical analysis used a one-way ANOVA with SPSS software (version 11.0).

## 3. Results

### 3.1. Cytotoxicity on MDA-MB-468 and Normal Vero Cell-Lines

The inhibitory effects, of cotyledon and pericarp extracts of* V. diospyroides* type SS fruit, on MDA-MB-468 and normal Vero cell-lines were evaluated by MTT and GFP assays, respectively. An overview of the results shows moderate to very strong antiproliferative (IC_50_ ≤ 20 *μ*g/mL) effects on MDA-MB-468 when this cell-line was cultured in the presence of any extract.  Table 1 lists the half-inhibitory IC_50_ concentrations of the extracts for 72-hour treatment. The acetone and methanolic extracts of cotyledon were the best chemopreventives against MDA-MB-468 cells based on their 1.60 and 3.50 *μ*g/mL IC_50_ values. Meanwhile, very strong inhibition by the acetone and methanolic extracts of pericarp was observed, with 17.45 and 10.64 *μ*g/mL IC_50_ values. The cytotoxicity against normal cells is shown in Table 2. All extracts were noncytotoxic with statistical significance, and the cell growth ranged between 88 and 100%. The extracts were inactive on normal cells and IC_50_ levels could not be determined. However, the acetone extract of cotyledon and the methanolic extract of pericarp at 50 *μ*g/mL concentrations would be toxic to normal cells, with 43.90 and 48.75 *μ*g/mL IC_50_ values. Although the 50% of inhibitions by* V. diospyroides* fruit extracts indicate successful antiproliferative activity against MDA-MB-468 breast cancer; this might be due to apoptosis and/or necrosis depending on the dose level [5]. Therefore, half IC_50_ dose levels of each extract were tested for their modes of cancer cell death and for times of therapeutic action.

### 3.2. Mode of Cancer Cell Death and Time of Therapeutic Action

**Figure 1:**
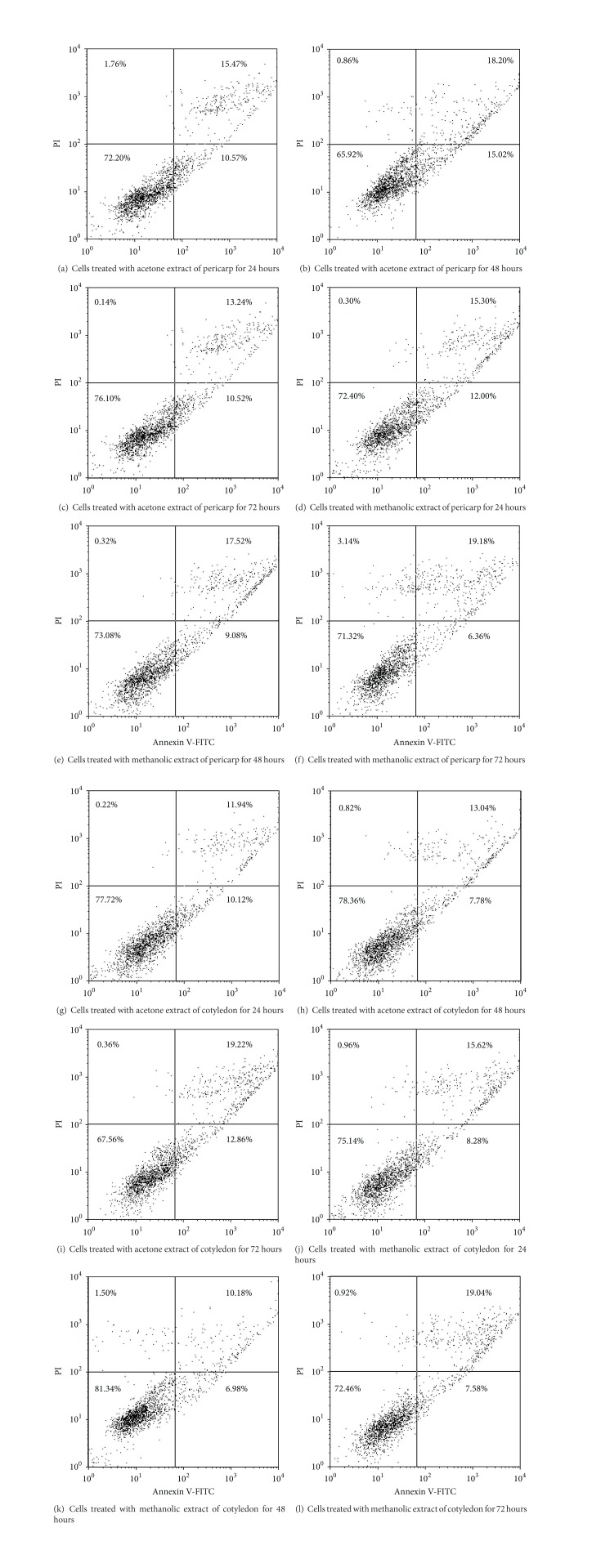
(a–l) Dot plots from FACS analysis of MDA-MB-468 cells treated with acetone extract of pericarp (a–c), methanolic extract of pericarp (d–f), acetone extract of cotyledon (g–i), and methanolic extract of cotyledon (j–l). All these extracts from* V. diospyroides* type SS fruit were used at half IC_50_ dose level and compared across a range of treatment times (24–72 h).

Time experiments on the therapeutic action, assessing the population of MDA-MB-468 cells following an apoptotic pathway, were carried out by treating the cells at half IC_50_ concentration for 24–72 hours with each extract. After staining the treated cells with Annexin V-FITC/PI, the mode of cell death was determined from the transition paths of the cells in the dot plot diagrams from FACS. The diagrams of FACS results are shown in Figure 1. The plots are divided into four quadrants, so that viable cells fall into the lower left quadrant (Annexin V-negative, PI-negative), early apoptosis is at lower right (Annexin V-positive, PI-negative), late apoptosis is at upper right (Annexin V-positive, PI-positive), and the nonviable cells are at upper left (Annexin V-negative, PI-positive). The apoptotic and necrotic pathways are distinguished as follows: apoptosis is identified by a continuous trace of treated cell from viable to early-late apoptosis and further to nonviable cells, while a direct trace from viable to nonviable quadrant is labeled as necrosis [5]. As shown in Figures 1(a)–1(l), in most extract treatments the lower left quadrant had half or more of the total cells, considered viable. Interestingly, by the above criteria the apoptotic pathway but not the necrotic pathway was clearly indicated by the traces in these plots. At 48 hours of treatment, the lowest viable counts and the highest apoptotic counts (early + late apoptosis) were found in the treatment with acetone extract of pericarp (65.92 + 33.22%; see Figure 1(b)). However, at 72 hours of treatment, the acetone extract of cotyledon was the most effective with 67.56 + 32.08% of the cells apoptotic (Figure 1(i)). This indicates that the therapeutic action of acetone extracts of cotyledon and pericarp peaked in their effects at different treatment times. In the treatment with methanolic extracts of pericarp, the populations of viable and early apoptotic cells declined continuously during 24 to 72 hours of treatment, from 72.40 to 71.32% and from 12.00 to 6.36%, respectively (see Figures 1(d)–1(f)). Simultaneously, in the same treatment, the populations of late apoptotic and nonviable cells increased from 15.30 to 19.18% and from 0.30 to 3.14%, respectively. On the other hand, over 24–72 hours of treatment, the cells treated with methanolic extract of cotyledon underwent apoptosis with only moderate numbers of nonviable cells and apoptotic cells (early + late apoptosis) (Figures 1(j)–1(l)).

**Figure 2:**
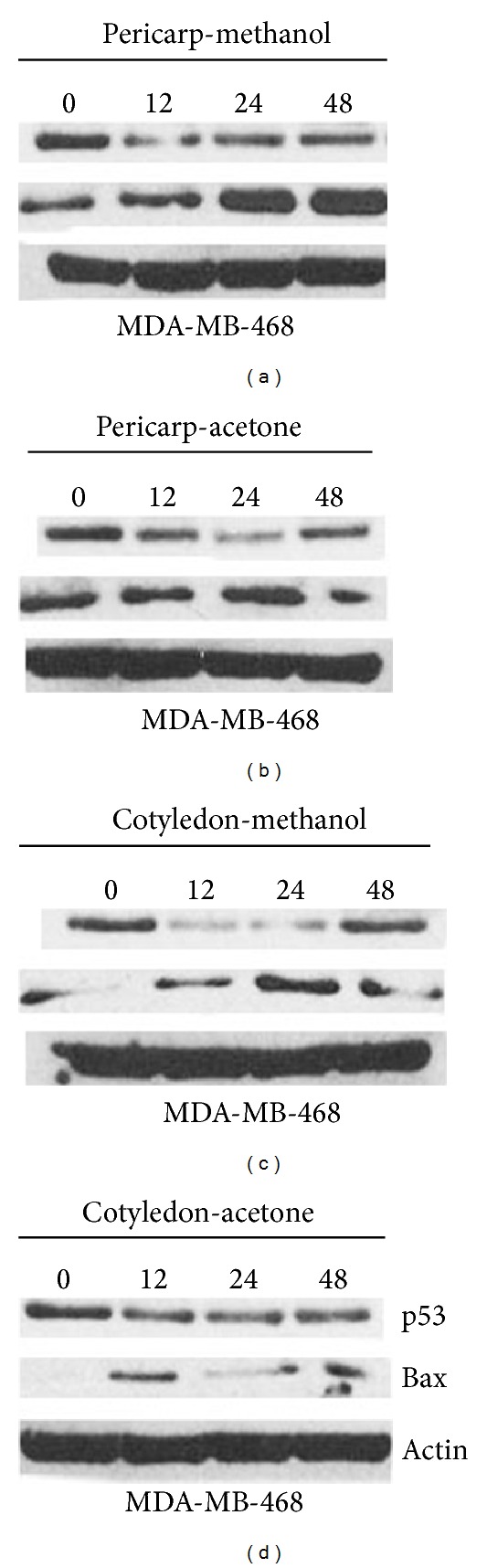
Effect of* V. diospyroides *type SS fruit on expression of the tumor suppressor protein p53 and proapoptotic protein, namely, the Bax. (a-b) MDA-MB-468 cells were treated with the extract of fruit pericarp, whereas (c-d) the cells were treated with the extract of fruit cotyledon, all at concentration of half IC_50_. The cellular proteins were separated on SDS-polyacrylamide gels and subsequently transferred to membranes. The protein levels were determined by western blots with appropriate antibodies directed against each protein.

Interestingly, in western blotting analysis, the expression of tumor suppressor protein (p53) and proapoptotic protein (Bax) confirmed that the fruit extracts of* V. diospyroides* could induce apoptosis pathway of MDA-MB-468 cells in time-dependent responses (12–48 hours). Although, there was detectable p53 protein expressed in all treated cells and times, continuous changes in the expression levels of Bax were detected in all treated cells, in a consistently time-dependent manner, indicating activation downstream of p53 (Figures 2(a)–2(d)). There were upregulations of Bax at 12–48 h for methanolic extract of pericarp, at 12–24 h for acetone extract of pericarp and methanolic extract of cotyledon, and at 12 h for acetone extract of cotyledon. The expression of Bax protein will be motivated leading finally to apoptosis. Simultaneously, apoptosis in all treated cells was also induced by the Bax protein in a consistently time-dependent response (12–48 hours).

## 4. Discussion

A previous study has shown that the crude extracts of* V. diospyroides* type LS fruit have strongly* in vitro* activity against the ER-rich MCF-7 and the ER-poor MDA-MB-468 breast cancer cell-lines [6]. In the present work instead of the type LS we used extracts of* V. diospyroides* type SS fruit and observed remarkable effects against the viability of MDA-MB-468 human breast cancer cell-line associated with the induction of apoptosis. A higher cytotoxic activity against cancer cells, indicated by a lower IC_50_, is anticipated with active apoptosis. Our results suggest that all extracts from type SS fruit have cancer chemopreventive potential* in vitro,* inhibiting cell proliferation with moderate to very strong activity (IC_50_ < 20 *μ*g/mL) [12, 13]. Furthermore, testing against the normal Vero cell-line did not show cytotoxicity; instead we observed cell growth >50% indicating that at IC_50_ the extracts might not inhibit normal cells. The various fruit parts and various organic solvents for extraction were explored. Acetone and methanolic extracts of cotyledon were the best in terms of cytotoxicity with 1.6 and 3.5 *μ*g/mL IC_50_ values. Both the more polar methanol and acetone have been widely used in the extraction of chemopreventive agents from various plant species.

In agreement with earlier results on the type LS fruit, acetone and methanol have been generally described as most suitable solvents for extraction that recover terpenoids, anthraquinones, and saponins in the crudes [6]. These phytochemicals have been investigated and shown as chemopreventives for various cancer therapies [15–17]. The crude extract of* V. diospyroides* type SS fruit might combine several active ingredients with antiproliferation effects on MDA-MB-468 cancer cells. On the other hand, the resveratrol tetramers vaticanol and vaticaphenol A have been first purified from the stem of* V. diospyroides* and have therapeutic properties that are documented against various cancers [7]. Subsequently resveratrol as a nontoxic agent has shown antineoplastic activity and very high efficacy in various cancer therapies [18–20]. Therefore, the therapeutic effects of* V. diospyroides* type SS fruit against MDA-MB-468 cells might be caused not only by the phytochemicals but also by the resveratrol compounds.

Most anticancer agents with minimal or absent side effects on normal cells inhibit cancer cell proliferation by cytotoxicity and activation of apoptosis [21, 22]. Our fruit extracts from type SS* Vatica* inhibited the proliferation of both ER-rich MCF-7 and ER-poor MDA-MB-231 breast cancer cell-lines, along pathways that might depend on the dose level [5]. An extract dose level at half IC_50_ was effective and only activated apoptosis of MCF-7. In contrast, death by necrosis was rapidly induced by acute toxicity at IC_50_ or higher dose levels. Apoptosis is precisely indicated by the loss of plasma membrane phospholipid asymmetry, with enzymatic cleavage of the DNA into oligonucleosomal fragments, and the subsequent segmentation of a cell into apoptotic bodies [23]. This is an important defensive mechanism against cancer progression with successes in cancer treatment [2]. Therefore, to avoid acute toxicity and side effects typical with a high dose level, low dose efficacy is critical to cancer chemopreventive agents. In the present work, half IC_50_ doses of the fruit extracts of* V. diospyroides* type SS were highly effective* in vitro* and induced apoptosis of breast cancer cell-lines.

Several techniques, including immunohistochemistry, western blots, and FACS, are known to elucidate the progression and mechanisms and discriminate between various types of cell death. By FACS analysis, various events, such as the blocking of cell cycle, activation of apoptotic genes or proteins, and loss of plasma membrane phospholipid asymmetry, can be detected and quantified to determine the apoptosis pathway [23]. Using live cancer cells without fixation, plasma membrane changes associated with apoptosis have been detected with Annexin V-FITC/PI bindings that represent apoptotic exploration and by monitoring the location of phosphatidylserine (PS) and integrity/permeability of cell membranes after treatment [5]. We investigated the apoptosis of breast cancer cells treated with extracts of type SS fruit, by Annexin V-FITC/PI binding assay using FACS method. After the FACS analysis, the traces of MDA-MB-468 cells treated at half IC_50_ concentration are shown in dot plots by symbol “7,” and criteria earlier described by us were applied to label the type of cell death [5]. The fruit extracts significantly induced apoptosis without necrosis in the MDA-MB-468 cells. Interestingly, with 48–72 hours of treatment, the acetone extracts of cotyledon and pericarp gave the highest fractions of apoptotic cells and the lowest fractions of viable cells. On the other hand, with a methanolic extract of pericarp, a continuous inhibition response to treatment time was observed. The viable cells and early apoptotic cells decreased consistently from 24 to 48 to 72 hours of treatment, while the counts of late apoptotic and nonviable cells increased. This indicates long-term therapeutic effects, not present with the acetone extract. Also, for the efficacy of therapeutic effects, at least 48 hours of treatment with the methanolic extract of cotyledon are required. These results confirm that the various extracts had different basic pharmacokinetic actions, in terms of both cytotoxic dose levels and dosing time effects.

The detailed mechanisms of apoptosis cannot be fully determined with an Annexin V-FITC/PI binding assay. Those mechanisms that involve the tumor suppressor protein (p53) and its products are still under study in several laboratories [2]. The p53 protein promotes apoptosis by proapoptotic and antiapoptotic pathways involving the Bcl2-family, whereas lack of p53 protein increases the risk of tumor emergence. The target of p53 in the Bcl-2 family is the Bax protein, a proapoptotic protein that promotes the release of cytochrome *c* from mitochondria inducing apoptosis [23]. In the present study, although mutant p53 is commonly known, the expressions of p53 and Bax protein are closely related to the dynamics of cells in apoptotic pathway detected by FACS method. Twelve to twenty-four hours of dosing with acetone extract of cotyledon and pericarp and methanolic extract of cotyledon gave the best results in terms of high percentage of apoptotic cells (up to 33%) and low percentage of living cells (down to 65%). On the other hand, treating with methanolic extract of pericarp in long period (more than 48 hours) could induce continuously apoptosis in time-dependent responses resulting in the highest percentage of dead cells (3.14%).

Therefore, p53 and Bax protein are now critical to the successful induction of apoptosis, and other key factors such as Bcl-2 protein, caspase-3, and PARP will be detailed in our future research on the MDA-MB-468 cells treated with* V. diospyroides* type SS fruit extracts.

## 5. Conclusions

The cotyledon and pericarp extracts of* V. diospyroides* type SS fruit significantly inhibited the ER-poor MDA-MB-468 breast cancer cell-line* in vitro*, even at half IC_50_. The mechanism of this therapeutic action was apoptosis induced during 48–72 hours of treatment, with appropriate dose depending on the type of extract used. The results suggest these extracts might support future clinical therapies, especially against both ER-rich and ER-poor breast cancers.

## Figures and Tables

**Table 1:** Half-inhibitory dose concentrations (IC_50_) of both acetone and methanolic extracts from *V. diospyroides* type SS fruit against MDA-MB-468 cell-lines.

Fruit part	Solvent	MDA-MB-468 cell	
		IC_50_, mean ± SD (*μ*g/mL)	Activity
Cotyledon	Acetone	1.60 ± 0.00^a^	Very strong
	Methanol	3.50 ± 0.60^a^	Very strong
Pericarp	Acetone	17.45 ± 2.10^c^	Moderate
	Methanol	10.64 ± 3.22^b^	Moderate

Statistically significant differences at *P* < 0.05 were determined by one-way ANOVA using SPSS version 11.0 program. Different superscripts (a, b, or c) indicate significant differences according to Tukey's multiple comparison test. Each result is based on three replicates.

**Table 2:** The acetone and methanol extracts of *V. diospyroides* type SS fruit at their IC_50_ concentrations (see Table 1) were inactive on normal Vero cells. The cell growth was assessed by a green fluorescent protein (GFP) assay.

Extract-fruit part	Extract concentrations (*μ*g/mL)	% Cell growth∗	Activity	IC_50_ (*μ*g/mL)
Acetone-cotyledon	50.00	26.53 ± 11.34^b^	Cytotoxic	43.90 ± 4.67
	16.67	102.74 ± 2.56^a^	Noncytotoxic	N/A
	5.56	104.17 ± 14.26^a^	Noncytotoxic	N/A
	1.85	108.71 ± 10.00^a^	Noncytotoxic	N/A
	0.62	107.94 ± 10.79^a^	Noncytotoxic	N/A
	0.21	110.78 ± 12.40^a^	Noncytotoxic	N/A

Acetone-pericarp	50.00	103.49 ± 32.03^a^	Noncytotoxic	N/A
	16.67	114.56 ± 7.96^a^	Noncytotoxic	N/A
	5.56	110.46 ± 11.19^a^	Noncytotoxic	N/A
	1.85	108.13 ± 10.80^a^	Noncytotoxic	N/A
	0.62	110.54 ± 5.94^a^	Noncytotoxic	N/A
	0.21	111.26 ± 6.65^a^	Noncytotoxic	N/A

Methanol-cotyledon	50.00	88.01 ± 15.42^a^	Noncytotoxic	N/A
	16.67	115.46 ± 11.87^a^	Noncytotoxic	N/A
	5.56	112.69 ± 8.84^a^	Noncytotoxic	N/A
	1.85	116.70 ± 11.63^a^	Noncytotoxic	N/A
	0.62	116.26 ± 11.07^a^	Noncytotoxic	N/A
	0.21	117.57 ± 7.79^a^	Noncytotoxic	N/A

Methanol-pericarp	50.00	25.34 ± 15.50^b^	Cytotoxic	48.75 ± 0.79
	16.67	112.68 ± 6.99^a^	Noncytotoxic	N/A
	5.56	104.09 ± 6.55^a^	Noncytotoxic	N/A
	1.85	104.45 ± 11.47^a^	Noncytotoxic	N/A
	0.62	104.64 ± 6.67^a^	Noncytotoxic	N/A
	0.21	104.89 ± 9.67^a^	Noncytotoxic	N/A

N/A means not applicable IC_50_ calculation.

-∗Cell growth reduced to less than 50% would indicate cytotoxicity against the Vero cells; otherwise an extract was considered inactive and the IC_50_ level could not be determined (N/A). Three independent replicates were performed.

-Different superscripts within a column indicate statistically significant differences in the Vero cell growth (mean ± SD) according to Tukey's multiple comparison test.

## References

[1] Jensen EV, Jacobson HI, Walf AA, Frye CA (2010). Estrogen action: a historic perspective on the implications of considering alternative approaches. *Physiology and Behavior*.

[2] Roy AM, Baliga MS, Katiyar SK (2005). Epigallocatechin-3-gallate induces apoptosis in estrogen receptor-negative human breast carcinoma cells via modulation in protein expresssion of p53 and Bax and caspase-3 activation. *Molecular Cancer Therapeutics*.

[3] World Health Organization Breast cancer: prevention and control. http://www.who.int/cancer/detection/breastcancer/en/index1.html.

[4] Granchi D, Cenni E, Savarino L (1998). Cell death induced by metal ions: necrosis or apoptosis?. *Journal of Materials Science: Materials in Medicine*.

[5] Srisawat T, Sukpondma Y, Graidist P, Chimplee S, Kanokwiroon K (2014). The dose dependent *in vitro* responses of MCF-7 and MDA-MB-231 cell lines to extracts of *Vatica diospyroides* Symington type SS fruit include effects on mode of cell death. *Pharmacognosy Magazine*.

[6] Srisawat T, Chumkaew P, Heed-Chim W, Sukpondma Y, Kanokwiroon K (2013). Phytochemical screening and cytotoxicity of crude extracts of *Vatica diospyroides* symington type LS. *Tropical Journal of Pharmaceutical Research*.

[7] Seo EK, Chai H, Constant HL (1999). Resveratrol tetramers from *Vatica diospyroides*. *Journal of Organic Chemistry*.

[8] Ito T, Akao Y, Yi H (2003). Antitumor effect of resveratrol oligomers against human cancer cell lines and the molecular mechanism of apoptosis induced by vaticanol C. *Carcinogenesis*.

[9] Dai JR, Hallock YF, Cardellina JH, Boyd MR (1998). HIV-inhibitory and cytotoxic oligostilbenes from the leaves of *Hopea malibato*. *Journal of Natural Products*.

[10] Sotheeswaran S, Diyasena MNC, Gunatilaka AAL, Bokel M, Kraus W (1987). Further evidence for the structure of vaticaffinol and a revision of its stereochemistry. *Phytochemistry*.

[11] Srisawat T, Chumkaew P, Maichum W, Sukpondma Y, Graidist P, Kanokwiroon K (2013). *In vitro* cytotoxic activity of *Vatica diospyroides* symington type LS root extract on breast cancer cell lines MCF-7 and MDA-MB-468. *Journal of Medical Sciences*.

[12] Alitheen NB, Mashitoh AR, Yeap SK, Shuhaimi M, Abdul Manaf A, Nordin L (2010). Cytotoxic effect of damnacanthal, nordamnacanthal, zerumbone and betulinic acid isolated from Malaysian plant sources. *International Food Research Journal*.

[13] Wibowo A, Ahmat N, Hamzah AS (2011). Malaysianol A, a new trimer resveratrol oligomer from the stem bark of *Dryobalanops aromatica*. *Fitoterapia*.

[14] Hunt L, Jordan M, De Jesus M, Wurm FM (1999). GFP-expressing mammalian cells for fast, sensitive, noninvasive cell growth assessment in a kinetic mode. *Biotechnology and Bioengineering*.

[15] Huang M, Lu J, Huang M, Bao J, Chen X, Wang Y (2012). Terpenoids: Natural products for cancer therapy. *Expert Opinion on Investigational Drugs*.

[16] Zhao J, Xu F, Huang H (2013). Evaluation on anti-inflammatory, analgesic, antitumor, and antioxidant potential of total saponins from *Nigella glandulifera* seeds. *Evidence-Based Complementary and Alternative Medicine*.

[17] Huang Q, Lu G, Shen H, Chung MCM, Choon NO (2007). Anti-cancer properties of anthraquinones from rhubarb. *Medicinal Research Reviews*.

[18] Aggarwal BB, Bhardwaj A, Aggarwal RS, Seeram NP, Shishodia S, Takada Y (2004). Role of resveratrol in prevention and therapy of cancer: preclinical and clinical studies. *Anticancer Research*.

[19] Kim SH, Kim HJ, Lee MH (2011). Resveratrol induces apoptosis of KB human oral cancer cells. *Journal of The Korean Society for Applied Biological Chemistry*.

[20] Li Y, Liu J, Liu X (2006). Resveratrol-induced cell inhibition of growth and apoptosis in MCF7 human breast cancer cells are associated with modulation of phosphorylated Akt and caspase-9. *Applied Biochemistry and Biotechnology*.

[21] Buolamwini JK (1999). Novel anticancer drug discovery. *Current Opinion in Chemical Biology*.

[22] Kaufmann SH, Earnshaw WC (2000). Induction of apoptosis by cancer chemotherapy. *Experimental Cell Research*.

[23] Elmore S (2007). Apoptosis: a review of programmed cell death. *Toxicologic Pathology*.

